# Effects of processing on the proximate and metal contents in three fish species from Nigerian coastal waters

**DOI:** 10.1002/fsn3.102

**Published:** 2014-03-19

**Authors:** Francisca I Bassey, Fehintola C Oguntunde, Chukwujindu M A Iwegbue, Vincent N Osabor, Christopher A Edem

**Affiliations:** 1 Department of Chemistry, University of CalabarCalabar, Cross-Rivers State, Nigeria; 2 Metals and Trace Organics Research Group, Department of Chemistry, Delta state UniversityP.M.B. 1, Abraka, Nigeria; 3 School of Chemistry and Physics, University of KwaZulu-NatalWestville Campus, Private Bag X54001, Durban, 4000, South Africa

**Keywords:** Culinary practices, dietary intakes, fish species, metals, target hazard quotient

## Abstract

The effects of culinary practices such as boiling, frying, and grilling on the proximate compositions and concentrations of metals (Cd, Pb, Cr, Zn, Fe, Cu, Mn, Ni, and Hg) in commonly consumed fish species from the Nigerian coastal waters were investigated. The selected fish species were *Polydactylus quadratifilis*, *Chrysicthys nigrodigitatus* and *Cynoglossus senegalensis*. The culinary practices lead to increased protein, fat, and ash contents and decreased moisture contents of these fish species. The culinary practices resulted significant increase in the concentrations of most of the studied metals and decrease in the concentrations of Fe, Cr, and Pb in some fish types. The concentrations and estimated dietary intakes of Cd, Pb, Cr, Zn, Fe, Cu, Mn, Ni, and Hg from consumption of the processed fish were within their statutory safe limits. The individual metal target hazard quotient (THQ) values and the total THQs were less than 1 which indicates that no health risks would arise from the long-term consumption of these fish species.

## Introduction

Fish constitute a major source of protein and healthy lipid for many throughout the world and its consumption has increased globally in the recent time. Fish provides the long-chain polyunsaturated omega-3 fatty acid which might favorably improve lipid profile and reduce cholesterol levels, the risk of coronary heart diseases, stroke and preterm diseases (Burger and Gochfeld 2005; Musaiger and D'Souza 2008; Bashir et al. 2012). In addition to this, fish consumption provides a good source of vitamins, minerals (Bashir et al. 2012) and other benefits which include their hypolipidemic/or antiatherogenic effects, decreased risk of prostate cancer, reduced occurrence of renal-cell carcinoma in women and reduced risk of dementia and Alzheimer disease in certain conditions (Musaiger and D'Souza 2008). Despite the human benefits from a fish diet, the levels of toxic contaminants in fish are of considerable concern because of the potential effects on the fish themselves and humans that consume them. Frequent fish consumption have been noted as a potential source of human exposure to toxic chemicals (Burger and Gochfeld 2005; Bashir et al. 2012).

The determination of metals in human foods is of significant concern due to their double-edged roles which range from the nutritional requirement of essential elements to the toxicity associated with the excessive intake of both the essential and toxic metals. Some metals such as Cd, Pb, and Hg are exceptionally toxic even at very low concentrations. For instance, Cd and Hg are known to impair kidney function, reproductive capacity, cause hypertension, tumor and hepatic dysfunction, and Pb can cause renal failure, liver damage, impaired hearing or cause mental retardation, while at elevated levels in women may result in a shortened gestation period (Iwegbue 2011). Metals such as Cu, Fe, Mn, and Zn are essential and required for normal body function such as the synthesis of metalloprotein. Deficiency of essential metals could lead to disease conditions. However, excessive intakes of these metals could cause toxicity problems which include premature aging and oxidative damage, a key component of chronic inflammatory disease and a suggested initiator of cancer (Naughton and Petroczi 2008).

Fish is a known source of some nutrients but cooking practices could cause modifications in its proximate composition, fatty and amino acids profiles, solubility and nutritional quality of fish. Although in most cases, fish are consumed in cooked form; the majority of studies related to the occurrence and the daily intake of metals through consumption fish are based on uncooked/raw product. Estimation of dietary intakes of metals based on the uncooked/or raw products could be misleading. However, several studies on the effect of culinary practices on heavy metal concentrations in fish and seafoods have been reported in the literature, for instance, in African catfish (Ersoy and Ozeren 2009; Ersoy 2011); sea bass (Ersoy et al. 2006), sea bass, and red seabream (He et al. 2010), sardine, hake, and tuna (Perello' et al. 2008); rainbow trout (Gokoglu et al. 2004), finfish and shellfish (Kalogeropoulos et al. 2012). In Nigeria, limited data are currently available on the effects of processing procedures on the proximate and metal compositions of Nigerian fishes. The objective of the present study was to determine the effects of cooking practices on the proximate and metal composition of some Nigerian fish species with a view to providing information on the health hazard associated with such culinary practices.

## Materials and Methods

### Sampling and sample preparation

Three fish species *Polydactylus quadratifilis, Chrysichthys nigrodigitatus* and *Cynoglassus senegalensis* were purchased from the Calabar Central Market. These fish species are among the popular types that are commonly consumed in southern Nigeria. The methods of cooking such as boiling, grilling, and frying are commonly practiced by the people. 2–4 kg of each type of fish comprising individuals of similar size were obtained. They were washed with cold water and the scales were removed with plastic knife, and prepared according to the Nigerian culinary practices. The fish was boiled for 45 mins at 120°C. The washed fish was fried in a frying pan which contained 470 mL of vegetable oil and was heated to a temperature of 170°C and until the fish turned brown. To achieve uniform cooking, the samples were occasionally turned by means of wooden spatula.

The three species were grilled in a domestic electric oven at 180°C. In order to avoid contact with metals, the metal grill was covered with grease proof paper on which a small hole had been opened to allow juice from the cooked food to drain. The fish was weighed before and after cooking in order to calculate water loss. The proximate analysis was carried out by using the Association of Official Analytical Chemists (AOAC) procedure. The moisture content was measured after drying in a vacuum oven at 100°C and dried to a constant weight (∼5 h). The protein content was measured by Kjeldahl methods which involves the conversion of protein and organic nitrogen to ammonia during digestion with sulfuric acid in the presence of mercury catalyst mixture. The acid digestion was made alkaline and the ammonia was distilled and titrated with standard acid. The percent nitrogen was determined and converted to protein using the 6.25. The analysis of fat was carried out by hydrolyzing the sample in water bath using 8 mol/L HCl after the addition of ethanol to liberate fat. The fat was extracted using ether and hexane. The extract was washed with diluted alkaline solution and filtered through a sodium sulfate column. The remaining extract was evaporated, dried, and weighed. Carbohydrate was obtained from the standard equation 100% − (%protein + %fat + %Ash + %moisture) and the energy content was obtained by multiplying the protein, carbohydrate, and fat content by the factors 4, 4, and 9, respectively. All samples were analyzed in triplicates and the concentrations were expressed in percentages (Musaiger and D'Souza 2008). For metal determination in the raw, boiled, grilled, and fried fish samples, 0.5 of the homogenized sample was weighed into a digestion tube where it was predigested using 10 mL concentrated HNO_3_ at 135°C until the liquid was clear, next, 10 mL of HNO_3_, 2 mL of H_2_O_2,_ and 2 mL of HClO_4_ were added and the temperature was maintained at 135°C for 1 h until the liquid become colorless. The digested liquors were filtered through Whatman no. 1 and diluted to 25 mL with water of 18.2 Ω cm (Millipore, Bellford, MA). For mercury determination, 2.0 g of the homogenized sample was weighed into digestion tube and 10 mL of concentrated HNO_3_ and 5 mL H_2_SO_4_ were slowly added. The tube was then placed on the top of a steam bath until complete dissolution was achieved. The tube was removed from the steam bath, cooled and the digest was carefully transferred into a 50 mL volumetric flask for the reduction of mercury using 5 mL of SnCl_2_.

### Chemical analysis

The determination of Cd, Pb, Ni, Cr, Cu, Fe, Mn, and Zn in the digested samples was carried out by using atomic absorption spectrophotometry (Perkin Elmer analyst 200, Norwalk, CT), while Hg was quantified by using cold vapor atomic absorption spectrometry (Varian VGA-77).

### Quality control/assurance

The quality control measures include the use of reagent and procedural blanks, sample duplicates and analysis of certified reference material (DORM-2) provided by NRC of Canada. The method blanks were carried for all elements by following the analytical steps in the analysis but omitting the sample. For every batch of 10 samples a reagent blank and a certified reference material were analyzed. The average blank reading was subtracted from instrumental reading before statistical analysis. The percentage recoveries of metals from the analysis certified reference material (DORM-2) are displayed in Table 1. Univariate treatments of the data were performed by analysis of variance (ANOVA) using statragraphic plus 400 (Statistical Graphic Corp., Herndon, VA). Duncan's multiple range tests were performed post hoc to evaluate differences among groups. Difference were considered statistically significant when *P* < 0.05.

**Table 1:** Validation method with standard reference material DORM-2 (Dogfish Muscle) (mg kg^−1^ dry weight)

Elements	DORM-2 (Dogfish Muscle) certified value	Measured value
Cd	0.043 ± 0.08	0.042 ± 0.04
Pb	0.065 ± 0.007	0.059 ± 0.06
Cr	34.7 ± 5.5	31.9 ± 4.8
Zn	25.6 ± 2.3	24.1 ± 2.7
Fe	142 ± 10	126.4 ± 8.2
Cu	2.34 ± 0.16	2.25 ± 0.21
Mn	3.66 ± 0.34	3.55 ± 0.28
Ni	19.45 ± 3.1	17.7 ± 3.6
Hg	4.64 ± 0.26	4.06 ± 0.31

### Estimation of dietary intake and target hazard quotients

The estimated daily intake (EDI) of metals from consumption of these fish species was evaluated using the formula;


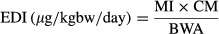
(1)

where MI is the mass of the fish ingested per day, CM is the concentration of metal in edible muscle of the fish, and BW is the body weight (60 kg).

The per capita fish consumption in Nigeria is 7.6 kg which is equivalent to 20.8 g of fish per day. To assess the level of concern arising from ingestion of metals from the consumption of these fish species, the target hazard quotient (THQ) values were calculated by using the measured concentrations of nine potentially toxic metals. The THQ is the ratio between measured concentration and oral reference dose, weighted by the length and frequency of exposure, amount ingested and body weight. The THQ was calculated by using the formula established by the United States Environmental Protection Agency (Naughton and Petroczi 2008).


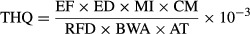
(2)

where EF is the exposure frequency (365 days/year), ED is the exposure duration (48.9 years) which corresponds to the average life expectancy of a Nigerian.

AT = averaging exposure time for noncarcinogens (365 days/year × ED). Note MI, CM and BWA have been previously defined in equation (1).The oral reference dose (mg/kg per day) used were, Cd (1 × 10^−3^), Pb (1.5), Cr (1.5), Zn (3.0 × 10^−1^), Fe (7.0 × 10^−1^), Cu (4.0 × 10^−2^), Mn (1.4 × 10^−1^), Ni (2 × 10^−2^) and Hg (5.0 × 10^−4^) (Naughton and Petroczi 2008).

## Results and discussion

### Proximate content

The proximate compositions of the raw and processed fish are displayed in Table 2. The proximate compositions of the fish samples are important for determining if the nutritive value conforms to the range of dietary requirement and commercial specifications. The protein contents of these fish species showed significant difference. The highest protein contents were observed in *C. senegalensis* (17.91%) while the lowest protein content was observed in *C. nigrodigitatus*. The protein contents in these three fish species followed the order: *C. Senegalensis* > *P. quadratifilis* > *C. nigrodigitatus*. Pierestani et al. (2009) reported the percentage of protein in fish species from the Caspian seas as 18.4–21.9%. Musaiger and D'Souza (2008) reported protein contents of 22.8% to 27.9% in fish consumed in the Arabian Gulf which are similar to the protein contents reported in this study. High protein contents indicate that essential amino acids composition of the fish is of a higher quality. The difference in the protein contents of these fish may be due to the differences in the level of assimilation of essential nutrients from their diets or environment and the conversion rates of these nutrients for the various metabolic activities.

**Table 2:** Proximate composition of raw and processed fish samples (%)^1^

Fish sample	Moisture (%)	Ash (%)	Protein (%)	Fat (%)	Carbohydrate (%)	Energy (%)
*Polydactylus quadratifilis*						
Raw	77.00 ± 3.60	1.28 ± 0.07	15.79 ± 1.23	4.08 ± 0.21	1.85	107.28
Boiled	72.50 ± 2.78	1.47 ± 0.12	17.86 ± 0.86	4.26 ± 0.10	3.91	125.42
Grilled	61.47 ± 2.54	2.25 ± 0.11	28.05 ± 1.99	6.31 ± 0.15	1.92	176.67
Fried	56.33 ± 3.04	2.40 ± 0.13	34.93 ± 1.30	7.35 ± 0.14	0.00	213.30
*Chrysicthys nigrodigitatus*						
Raw	74.47 ± 0.98	1.28 ± 0.04	12.10 ± 1.28	4.29 ± 0.25	7.86	216.45
Boiled	71.27 ± 1.37	1.64 ± 0.06	17.10 ± 1.57	4.50 ± 0.65	5.49	130.86
Grilled	58.00 ± 2.08	4.03 ± 0.12	24.36 ± 0.45	6.37 ± 0.75	7.24	183.19
Fried	51.40 ± 3.23	4.23 ± 0.13	26.61 ± 0.88	8.12 ± 0.61	10.41	214.23
*Cynoglossus senegalensis*						
Raw	77.40 ± 1.71	1.27 ± 0.11	17.61 ± 1.93	3.35 ± 0.19	1.09	113.39
Boiled	71.33 ± 0.94	1.69 ± 0.10	20.68 ± 1.68	3.99 ± 0.27	2.31	127.87
Grilled	61.70 ± 2.04	3.27 ± 0.17	24.20 ± 0.88	6.10 ± 0.63	4.28	168.82
Fried	56.97 ± 2.01	4.18 ± 0.14	28.66 ± 2.91	7.49 ± 0.28	2.70	192.85

1 Results are expressed as mean ± SD of three determinations.

The highest fat content was observed in *C. nigrodigitatus,* while the lowest fat content was observed in *C. senegalensis* that is it followed the order: *C. nigrodigitatus* > *P. quadratifilis* > *C. senegalensis*. The results showed a reversed order of that of the protein content which indicates that low-fat fish contain higher amounts of protein. The fat contents in these fish species were similar to 3.50–8.25% found in some freshwater fish species (Fawole et al. 2007). The fat content is a reflection of the fact that fish contains essential fatty acids needed for prevention of cardiovascular disorder as well as maintaining proper functioning of membrane in living cells.

The ash and moisture contents of these fish species varied over a very small range. However, the moisture contents of these fish species follow the order: *C. senegalensis* >*P. quadratifilis* > *C. nigrodigitatus* which was the reversed order of fat contents. This is in line with the fact that low-fat fish species have higher water contents and as a result, their flesh is usually whitish in color (Pierestani et al. 2009). For example, Pierestani et al. (2009) reported that *Liza aurata, Caprinus carpio* and *Sander Lucioperca* had fat contents lower than 5% and were classed as low/lean fish with moisture contents above 75%, while *Clupeonella cultriventris caspia* and *Ritilus frisii kutum* were classed as fat rich fish with moisture contents of below 74.9% and fat contents above 5%. The highest carbohydrate content was found *C. nigrodigitatus,* while the lowest carbohydrate was found in *C. senegalensis*.

### Effect of processing in the proximate composition

The proximate composition of these fish was significantly influenced by the processing methods (boiling, grilling and frying). As shown in Table 2, the protein increased significantly (*P* < 0.05) in the order frying > grilling > boiling. Loss of moisture during processing could lead to concomitant increase in the protein content. However, frying had the greatest moisture losses compared with grilling or boiling and therefore had the highest amounts of protein. This is in accordance with the findings of Gokoglu et al. (2004) and Kocatepe et al. (2011) that fried and microwave-cooked fish had significantly higher protein than raw fish. Musaiger and D'Souza (2008) reported similar trend on the effects of cooking methods on the proximate composition of fish and shrimp. The various cooking methods significantly influenced the fat contents of these fish species. During boiling the fat contents of these fish types increased 4.4% to 19.1%. The highest percent increase in the fat content during boiling was observed *C. senegalensis* and the lowest percent increase was observed in *P. quadratifilis*. Grilling lead to ∼48.5% to 82% increase in the fat contents of these species, while frying gave 80.1% to 123.6% increase in the fat contents of these species. Increase in the fat contents of these fish species during frying could be related in part to the absorption of oil and partial loss of water by evaporation (Musaiger and D'Souza 2008). The trends observed in the results of the present study were similar to those reported in the literature for boiled and fried common *Silver barb*, *Nile tilapia*, *Walking catfish*; in boiled and steamed *Striped catfish,* in fried Spanish mackerel (Puwastien et al. 1999), fried, oven-baked, and grilled in *Sardina pilchardus* (García-Arias et al. 2003), in oven-cooked and microwave cooked *Oncorhynchus mykiss* (Unusan 2007), in fried, baked, and microwave-cooked *Dicentrarcus labrax* (Türkkan et al. 2008), in fried *Clarias gariepinus* (Ersoy and Ozeren 2009) and in grilled, fried, and microwave-cooked archovy (Kocatepe et al.,^2011^). The levels of oil absorption during frying vary from one fish type to another. In this study, *C. senegalensis* had the greatest oil absorption rate during frying. Like protein and fat contents, the ash contents of fish species were also influenced in similar direction by the processing methods. For example, boiling lead to 14.8% to 33.1% increase in the ash contents of these fish species, grilling gave 75.8% to 214.8%, while frying gave 87.5% to 229.1% increase in the ash contents of these fish species. Ersoy and Ozeren (2009) reported increase in the ash content of African catfish during grilling and frying The percent increase in the ash contents of these fish species under different cooking methods follow the order: *C. senegalensis* >*C. nigrodigitatus* > *P. quadratifilis*.

In this study, the moisture contents of these fish species decreased during processing. The decrease in moisture contents followed the order frying > grilling > boiling. The percentage decrease in moisture contents during processing ranged from 4.3% to 7.8%, 20.2% to 22.1%, and 22.9% to 30.1% for boiling, grilling, and frying, respectively. The moisture loss in these fish species followed the order: *C. nigrodigitatus* > *C. senegalensis* > *P. quadratifilis*. Grilling and frying were carried at high temperatures which could lead to significant loss of water from the fish species. The reduction in moisture is advantageous as it could lead to reduction in the fish susceptibility to microbial spoilage, oxidative degradation of poly unsaturated fatty acid, and consequently improves the quality of the fish and also enables longer preservation (Frankel 1991). In this study, the different culinary methods caused significant increase in the energy contents of these fish species than the raw samples except for *C. nigrodigitatus* in which culinary procedure caused a decrease in the energy content. Kocatepe et al. (2011) reported that cooking methods such as grilling, frying and microwaves cooking caused a decrease in the percent carbohydrate content of archovy, while baking caused increase in the carbohydrate content. In this study, the carbohydrate contents found in *P. quadratifilis* were similar to the carbohydrate content of archovy, while that of *C. nigrodigitatus* and *C. senegalensis* were higher than that of archovy.

### Metal concentrations of the raw and processed fish

The metal concentrations of the raw and processed fish samples are displayed in Tables 3, 4, respectively. There is no difference (*P* < 0.05) in the concentrations of Cd in the three fish species. The concentrations of Cd observed in these fish species (raw and processed) were below the permissible limit of 0.2 mg kg^−1^ (MAFF 1995). Alinor and Obiji (2010) reported Cd concentrations in the range of 0.019 to 0.062 mg kg^−1^ in *Tilapia guineensis*, *Liza grandisaquamis*, *Synaptura insitanica* and *Sphyraena sphyraena* from Nworie River, Nigeria. Frias-Esperiicueta et al. (2010) reported mean Cd levels of 0.27–0.32 mg kg^−1^ in four commercial fish species of New Mexico. The processing methods had significant impact on the Cd concentrations in these fish species except *C. senegalensis*. In *P. quadratifilis*, boiling and frying lead to 100% increase in the Cd concentration, while grilling gave seven times the concentration of the Cd in the raw fish. Also, grilling gave two times increase in the concentration of Cd in *C. nigrodigitatus*. The increment of Cd during grilling and frying was obviously the result of water loss (Kalogeropoulos et al. 2012).

**Table 3:** Metal concentrations in mg kg^−1^ of the raw fish species^1^

	M ± SD		
	
Heavy metals	*Polydactylus quadratifilis*	*Chrysicthys nigrodigitatus*	*Cynoglossus senegalensis*
Cd	0.010 ± 0.005	0.010 ± 0.004	0.010 ± 0.003
Pb	0.034 ± 0.012	0.046 ± 0.007	0.053 ± 0.004
Cr	0.006 ± 0.16	0.011 ± 0.06	0.017 ± 0.03
Zn	0.450 ± 0.05	0.660 ± 0.12	1.331 ± 0.14
Fe	0.550 ± 0.06	1.750 ± 0.24	1.720 ± 0.14
Cu	0.210 ± 0.07	0.650 ± 0.07	0.122 ± 0.07
Mn	0.009 ± 0.04	0.146 ± 0.04	0.480 ± 0.23
Ni	0.009 ± 0.08	0.011 ± 0.03	0.013 ± 0.00
Hg	0.010 ± 0.00	0.010 ± 0.001	BDL

BDL, below detectable limit.

1 Results are expressed as mean ± SD of three determinations.

**Table 4:** Influence of processing on metal contents in mg kg^−1^ of the investigated fish species^1^

	*Polydactylus quadratifilis*			*Chrysicthys nigrodigitatus*			*Cynoglossus senegalensis*		
			
	Boiled	Grilled	Fried	Boiled	Grilled	Fried	Boiled	Grilled	Fried
Cd	0.020 ± 0.003	0.070 ± 0.009	0.020 ± 0.014	0.010 ± 0.002	0.020 ± 0.001	0.010 ± 0.002	0.010 ± 0.005	0.010 ± 0.00	0.010 ± 0.002
Pb	0.036 ± 0.007	0.058 ± 0.00	0.48 ± 0.001	0.031 ± 0.011	0.048 ± 0.013	0.047 ± 0.012	0.042 ± 0.002	0.041 ± 0.005	0.042 ± 0.007
Cr	0.011 ± 0.19	0.013 ± 0.05	0.012 ± 0.11	0.007 ± 0.02	0.013 ± 0.04	0.010 ± 0.04	0.014 ± 0.01	0.012 ± 0.07	0.018 ± 0.06
Zn	0.670 ± 0.13	0.880 ± 0.10	0.860 ± 0.09	1.650 ± 0.16	1.201 ± 0.23	1.100 ± 0.09	0.048 ± 0.11	1.263 ± 0.13	1.450 ± 0.09
Fe	1.510 ± 0.43	1.570 ± 0.29	1.650 ± 0.10	1.022 ± 0.32	3.502 ± 0.29	3.830 ± 0.23	0.075 ± 0.32	1.690 ± 0.19	1.850 ± 0.09
Cu	0.300 ± 0.11	0.290 ± 0.36	0.280 ± 0.16	0.111 ± 0.10	0.403 ± 0.12	0.180 ± 0.05	0.143 ± 0.09	0.300 ± 0.03	0.320 ± 0.06
Mn	0.013 ± 0.07	0.210 ± 0.32	0.200 ± 0.14	0.102 ± 0.09	0.172 ± 0.04	0.191 ± 0.01	0.160 ± 0.26	0.262 ± 0.20	0.413 ± 0.19
Ni	0.010 ± 0.13	0.009 ± 0.03	0.009 ± 0.02	0.010 ± 0.01	0.031 ± 0.08	0.55 ± 0.05	0.007 ± 0.004	0.027 ± 0.002	0.035 ± 0.004
Hg	0.009 ± 0.001	0.016 ± 0.001	0.010 ± 0.004	0.010 ± 0.004	0.013 ± 0.002	0.10 ± 0.006	BDL	BDL	BDL

BDL, below detectable limit.

1 Results are expressed as mean ± SD of three determinations.

The highest mean level of Pb was observed in *C. senegalensis* and there was significant difference in Pb concentrations in these fish species. The permissible limit of Pb in fish is set at 2.0 mg kg^−1^ (MAFF 1995). The concentrations of Pb in the raw and the processed fish were below the permissible limit. Lead concentrations of 0.8–1.1 mg kg^−1^ were recorded for marine fin fish in Langkowi Island, Malaysia (Irwandi and Farida 2009). Bashir et al. (2012) reported Pb contents of 0.12–0.13 mg kg^−1^ in muscles of *Arius thalassinus* and *Pennahia anea* from Kapar and Mersing coastal waters in Malaysia. Mean Pb levels of 2.14 mg kg^−1^ was found in *C. carpio* samples from Avsar Dam Lake, Turkey (Ozturk et al. 2009). Alinor and Obiji (2010) reported Pb contents ranging from not detected to 0.58 mg kg^−1^ in *Tilapia guineensis*, *Liza grandisaquamis*, *Synaptura insitanica,* and *Sphyraena sphyaena* from Nworie River, Nigeria. Ubalua et al. (2007) reported Pb levels between 0.01 and 0.03 mg kg^−1^ in shellfish and fish in Aba River, Nigeria. Frias-Esperiicueta et al. (2010) reported mean Pb contents in the range of 2.12–2.80 mg kg^−1^ in four commercial fish species from New Mexico. During processing, the concentration of Pb in *P. quadratifilis* increased by 5.9% for boiling, 70.6% for grilling, and 41.2% for frying. Boiling caused 32.6% decrease in the Pb concentration in *C. nigrodigitatus,* while frying and grilling caused 2.2% and 4.2% increase in Pb concentrations, respectively. The processing methods caused a decrease in the concentration of Pb in *C. senegalensis*. For instance, the boiled, grilled, and fried *C. senegalensis* decreased in its Pb contents by 20.8%, 22.6% and 20.8%, respectively. There was no significant difference in the mean concentrations of Pb in the boiled, fried, and grilled *C. senegalensis*.

In this study, *C. senegalensis* had higher concentrations of Cr than the other fish species. The Brazilian permissible limit for Cr in fish was set at 0.1 mg kg^−1^ (Tarley et al.^2001^). The concentrations of Cr observed in raw and processed samples of these fish species were below the permissible limit. Burger and Gochfeld (2005) reported Cr concentrations of 0.03 to 0.34 mg kg^−1^ in commercial fish from New Jersey Market, USA. The processing method had significant effects on the concentrations of Cr in *P. quadratifilis*. Boiling increased the Cr concentration by 183.3%, grilling by 216.7%, and frying by 200%. However, in *C. nigrodigitatus* boiling caused a significant decrease in Cr concentration (36.4%), while grilling gave 18.2% increase and no increase in Cr was observed with frying. In *C. senegalensis* boiling and grilling caused 17.6% to 29.4% decrease in Cr, while frying lead to 5.9% increase in Cr concentration. The increase during frying could be due to moisture loss and uptake of Cr from the oil during frying.

The highest zinc concentration was observed in *C. senegalensis,* while the lowest concentration was found in *P. quadratifilis*. The permissible limit of Zn in fish was set at 50 mg kg^−1^ (MAFF 1995). The concentrations of Zn in the raw and the processed fish samples were below the permissible level of Zn in fish. The impact of processing on the Zn contents of these fish species varied significantly. In *P. quadratifilis* and *C. nigrodigitatus* boiling, grilling, and frying caused significant increase (48.9% to 150%) in the concentration of Zn, whereas in *C. senegalensis,* boiling and grilling caused 96% and 5.1% decrease in the Zn concentration while frying gave 9.6% increase in Zn concentration. In *C. nigrodigitatus,* the Zn concentrations followed the order: boiled > grilled > fried, the reverse of this order was observed in *P. quadratifilis*.

The concentrations of Fe in raw and processed fish samples varied significantly with the highest mean concentration was observed in *C. nigrodigitatus,* while the lowest mean Fe concentration was observed in *P. quadratifilis*. Frias-Esperiicueta et al. (2010) reported mean iron concentrations of 7.53–20.66 mg kg^−1^ in *Mugil cephalus*, *Diapterus sp., Oreochromis aureus* and *Scomberomorus sierra* from Mexico. Alinor and Obiji (2010) reported Fe concentrations in the range of 3.10–4.76 mg kg^−1^ in fish samples from Nworie Rivers, Nigeria. Iron concentration of 16.55 mg kg^−1^ was found in muscles of *C. carpio* samples from the Avsar Dam Lake (Ozturk et al. 2009). The concentrations of Fe as observed in our study were lower than concentrations reported by these researchers. The processing methods showed different effects on the different fish species. During processing, the concentration of Fe in *P. quadratifilis* increased by 174–200%. Fried *P. quadratifilis* had the highest concentration of Fe while the boiled *P. quadratifilis* had the lowest Fe concentration. In *C. nigrodigitatus*, processing gave 6% to 37% decrease in the concentrations of Fe. Grilling and frying gave 34% and 37% decrease in Fe concentrations, respectively. However, these cooking methods had no significant effect on the concentrations of Fe observed in *C. senegalensis,* only frying caused 7.6% increase in the concentration of Fe.

The highest Cu was observed in *C. nigrodigitatus,* while the lowest Cu was observed in *P. quadratifilis*. The concentrations of Cu in the processed fish samples ranged between 0.111 and 0.403 mg kg^−1^. The concentrations of Cu in the raw and processed fish were below the permissible limit (20 mg kg^−1^) of Cu in fish (MAFF 1995). Processing caused significant increase in the concentrations of Cu in *P. quadratifilis* (33.3–42.9%) and *C. senegalensis* (17.2–162.2%) while processing caused decrease in Cu concentrations observed in *C. nigrodigitatus* (38–82%).

The highest concentration of Mn was observed in *C. senegalensis* while the lowest concentration was observed in *P. quadratifilis* (Table 3). The effect of the various processing methods on the concentrations of Mn varied from one fish type to another. For example, processing caused 44% to 223% increase in Mn concentration of *P. quadratifilis,* while grilling and frying caused 17.8% and 30.8% increase in Mn concentration in *C. nigrodigitatus,* and boiling caused 30.1% decrease in the Mn in *C. nigrodigitatus*. However, in *C. senegalensis,* processing caused a significant decrease in Mn concentration (14.0–66.7%). The decrease in Mn concentrations during processing of *C. senegalensis* followed the order: boiled > grilled > fried.

The concentrations of Ni in the three fish species were similar and ranged between 0.009 and 0.013 mg kg^−1^, while Ni concentrations ranging from 0.007 to 0.55 mg kg^−1^ were observed in the processed fish samples. Ozturk et al. (2009) reported mean Ni content of 1.27 mg kg^−1^ in muscles of *C. carpio* from the Avsar Dam Lake. The processing methods had no significant effect on the concentrations of Ni in *P. quadratifilis,* whereas grilling and frying caused significant increase in Ni concentrations in *C. nigrodigitatus* and *C. senegalensis*. Boiling caused a decrease in Ni concentrations of *C. nigrodigitatus* and *C. senegalensis*. Mercury was found in these three fish species at concentration ranging from not detected to 0.010 mg kg^−1^. There was no significant difference in Hg concentrations in *P. quadratifilis* and *C. nigrodigitatus*. In *C. senegalensis*, the concentrations of Hg in the raw and processed were below the detection limit in the raw and processed fish. The permissible limit for Hg in fish is set at 0.5 mg kg^−1^ (MAFF 1995). The concentration of Hg in the raw and the processed fish samples were below the permissible limits. Burger and Gochfeld (2005) reported Hg concentrations ranging from 0.01 to 0.65 mg kg^−1^ in commercial fish from New Jersey market. Irwandi and Farida (2009) reported Hg concentration between not detected and 0.08 mg kg^−1^ in marine fin fish in Langkwi Island, Malaysia. In the literature, there are contradictory reports on the relationship between trace metal contents of raw and cooked fish. For example, Kalogeropoulos et al. (2012) reported that metal concentrations in cooked fish and shellfish were in all cases higher than raw samples, following the pattern: pan fried > grilled > raw, the increment in most cases is statistically significant. Ersoy (2011) reported that microwave heating of African catfish caused a decrement in the Cr content but has no effect on the Pb concentration while frying resulted in decreased Zn concentration and elevated Cu concentration in rainbow trout (Gokoglu et al. 2004). Perello' et al. (2008) reported that the concentrations of As, Hg, and Pb in the fish samples (sardine, hake and tuna) showed a clear tendency, in general, to increase after cooking. However, in these samples, Cd levels were very close to their detection limit. Ersoy et al. (2006) reported that baking caused a decrease in the Pb concentration of sea bass while frying caused an increase in the concentration of As. Gall et al. (1983) observed no significant change in concentrations of Zn, Cu, and Fe after cooking fish fillet. The possible explanation of the contradictory results reported in the literature could be associated with the size of the cooked fish, and interplay between the size, oil uptake and water loss during frying and grilling processes as well as metal evaporation during these processes.

### Estimation of dietary intake and THQ

It is pertinent to note that concentrations of metals in the processed fish gives a better estimate of the dietary intake of metals than the raw fish since fish are not consumed in their raw form. The estimated dietary intakes of metals based on per capital consumption of 7.6 kg fish are displayed in Table 5 while Table 6 provided information on the THQ of metals from consumption of these fish species. An important aspect in assessing risk to human health from potentially toxic chemical in food is the knowledge of dietary intake of such substance in comparison with the safety margin (Kalogeropoulos et al. 2012).

**Table 5:** Estimated daily intake of metals (*μ*g kg^−1^ bw day^−1^) from consumption of 20.8 g of fish per day

	*Polydactylus quadratifilis*			*Chrysicthys nigrodigitatus*			*Cynoglossus senegalensis*			
				
Metal	Boiled	Grilled	Fried	Boiled	Grilled	Fried	Boiled	Grilled	Fried	Permissible tolerance intakes (PTI) per day/*μ*g per day/60 kg body weight
Cd	0.007	0.02	0.007	0.004	0.007	0.004	0.004	0.004	0.004	0.35^1^
Pb	0.013	0.02	0.016	0.011	0.016	0.016	0.015	0.014	0.015	3.6^2^
Cr	0.004	0.005	0.004	0.003	0.005	0.004	0.005	0.004	0.006	200^3^
Zn	0.23	0.32	0.30	0.57	0.42	0.38	0.02	0.48	0.502	12000^4^
Fe	0.52	0.54	0.57	0.354	1.214	1.328	0.61	0.59	0.64	12500^4^
Cu	0.10	0.10	0.10	0.038	0.14	0.06	0.05	0.10	0.11	5000^5^
Mn	0.005	0.073	0.069	0.035	0.060	0.066	0.055	0.09	0.143	–
Ni	0.003	0.003	0.003	0.003	0.011	0.191	0.002	0.009	0.012	12^6^
Hg	0.003	0.006	0.003	0.003	0.005	0.003	–	–	–	1

1 EFSA (2011).

2 WHO (2003).

3 RDA (*μ*g/day).

4 RDI (*μ*g/day) (NRC (National Research Council) 1989); ^5^TDI *μ*g/day (EU 2003).

6 WHO (2008).

**Table 6:** Target hazard quotient for consumption of 20.8 g per day

	*Polydactylus quadratifilis*			*Chrysicthys nigrodigitatus*			*Cynoglossus Senegalensis*		
			
	Boiled	Grilled	Fried	Boiled	Grilled	Fried	Boiled	Grilled	Fried
Cd	0.007	0.002	0.007	0.004	0.007	0.004	0.004	0.004	0.004
Pb	0.000	0.000	0.000	0.00	0.000	0.000	0.000	0.000	0.000
Cr	0.000	0.00	0.000	0.000	0.000	0.000	0.000	0.000	0.000
Zn	0.00	0.001	0.001	0.002	0.001	0.001	0.000	0.002	0.002
Fe	0.001	0.001	0.001	0.001	0.002	0.002	0.001	0.001	0.001
Cu	0.001	0.003	0.003	0.001	0.004	0.000	0.001	0.003	0.003
Mn	0.003	0.001	0.000	0.000	0.000	0.000	0.000	0.001	0.001
Ni	0.000	0.000	0.000	0.000	0.001	0.010	0.000	0.000	0.001
Hg	0.006	0.012	0.006	0.006	0.010	0.006	–	–	–
∑THQ	0.018	0.038	0.018	0.014	0.025	0.023	0.006	0.011	0.012

THQ, target hazard quotient.

The EDI of Cd from consumption of these processed fish species ranged between 0.004 *μ*g kg^−1^ bw day^−1^ and 0.02 *μ*g kg^−1^ bw day^−1^ with the highest Cd intake coming from the consumption of grilled fish. The provisional tolerable weekly intake (PTWI) of Cd is set at 2.5 *μ*g kg^−1^ bw by the European Food Safety Authority (EFSA 2011), which is equivalent 0.35 *μ*g kg^−1^ bw day^−1^. The EDI constituted 1.1% to 5.6% of the provisional tolerable daily intake (TDI) of Cd. Kalogeropoulos et al. (2012) reported weekly intake of 0.02 to 1.28 *μ*g Cd kg^−1^ bw day^−1^ and 0.01 to 0.08 *μ*g Cd kg^−1^ bw day^−1^ from consumption of fried and grilled seafoods in Greece.

The estimated dietary intakes of Pb from consumption of these fish species were similar (i.e. 0.011 *μ*g kg^−1^ bw day^−1^ to 0.02 *μ*g kg^−1^ bw day^−1^). The PTWI of Pb is 25 *μ*g kg^−1^ bw (3.6 *μ*g kg^−1^ bw day^−1^ [WHO, 2003]). The estimated intake of Pb constituted 0.31% to 0.56% of the provisional daily intake of Pb. Kalogeropoulos et al. (2012) reported weekly intake values of 0.03–1.45 *μ*g Pb kg^−1^ bw and 0.05–0.35 *μ*g Pb kg^−1^ bw for consumption of fried and grilled seafood in Greece. The daily intakes of Cr in this study ranged from 0.003 to 0.006 *μ*g kg^−1^ bw day^−1^. This highest intake value for Cr was obtained from consumption of fried *C. senegalensis*. The recommended daily intake of Cr is 200 *μ*g/day. The EDI of Cr constituted 0.09% to 0.18% of recommended daily intake. The recommended daily intakes of Zn and Fe are set at 12000 and 12500 *μ*g day^−1^, respectively (NRC [National Research Council] 1989). In this study, the EDI of Zn and Fe from consumption of boiled, grilled food, fried fish ranged from 0.02 to 0.57 *μ*g kg^−1^ bw day^−1^ and 0.31 to 1.33 *μ*g kg^−1^ bw day^−1^, respectively. Higher Zn intakes were obtained from consumption of boiled *C. nigrodigitatus* while intakes of iron were obtained from consumption of grilled and fried *C. nigrodigitatus* as compared to other two species. The estimated dietary intakes of Zn and Fe in this study were below the recommended daily intake values. The EDI values of Cu and Mn ranged from 0.07 to 0.14 *μ*g kg^−1^ bw day^−1^ and 0.005 to 0.14 *μ*g kg^−1^ bw day^−1^, respectively. Higher dietary intakes of Mn were obtained from consumption of grilled and fried *C. senegalensis*. The TDI of Cu is 5000 *μ*g day^−1^ (which is equivalent to 83 *μ*g kg^−1^ bw day^−1^) (EU 2003). The NRC has recommended safe and adequate daily intake levels for Mn that range from 0.3 to 1 mg/day for children up to 1 year, 1–2 mg/day for children (1–3 year old) and males and females (19–70 years old) is 2 and 11 mg/day, respectively (Institute of Medicine 2003). The estimated intakes of Cu and Mn were below the recommended safe intake levels. The EDI of 0.003 to 0.19 *μ*g kg^−1^ bw day^−1^ Ni was recorded in this study which constituted 0.03% to 1.58% of TDI value of 12 *μ*g kg^−1^ bw day^−1^ (WHO 2003). In this study, the consumption of *C. senegalensis* contributed no Hg to the dietary intake, while Hg intakes of 0.003 to 0.006 *μ*g kg^−1^ bw day^−1^ were obtained from consumption of boiled, fried, and grilled *P. quadratiifilis* and *C. nigrodigitatus*). The provisional TDI of Hg is set at 1 *μ*g kg^−1^ bw day^−1^. The EDI of Hg constituted 0.3% to 0.6% of the provisional TDI value.

The interpretation of the THQ value is binary; THQ is either >1 or <1, where THQ > 1 indicates a reason for health concern. It must be noted that THQ is not a measure of risk but indicates a level of concern. The THQ values are additive not multiplicative, for example the level of concern at THQ of 20 is larger but not 10-fold of those at THQ = 2 (Naughton and Petroczi 2008). According to the data displayed in Table 5, individual metal had THQ values less than 0.02 and the combined THQ values (TTHQ) were less than 0.05. According to the guideline for interpretation of THQ, there is no health concern arising from the consumption of these cooked fish species. The THQ values reported in this study were lower than values reported for consumption of processed fish in Greece (Kalogeropoulos et al. 2012).

## Conclusion

The culinary practices such as boiling, grilling, and frying resulted in increased protein, fat, and ash contents and decreased moisture contents of the three fish species. The cooking methods caused significant increase in the concentrations of most metals compared to those of the raw samples. However, these culinary practices also caused a decrease in the concentrations of metals (Fe, Cr, and Pb) in some fish types. This behavior could be attributed to the inter-play between the size of the cooked fish, oil uptake, water loss, and metal evaporation during processing. The concentrations, estimated dietary intakes and THQ of the metals were within safe limits, indicating that no health concern would arise from the consumption of these fish species.
